# Surgery for Bowen Disease: Clinicopathological Factors Associated With Incomplete Excision

**DOI:** 10.5826/dpc.1102a46

**Published:** 2021-04-12

**Authors:** Julia Fougelberg, Hampus Ek, Magdalena Claeson, John Paoli

**Affiliations:** 1 Department of Dermatology and Venereology, Institute of Clinical Sciences, Sahlgrenska Academy, University of Gothenburg, Gothenburg, Sweden; 2 Region Västra Götaland, Sahlgrenska University Hospital, Department of Dermatology and Venereology, Gothenburg, Sweden; 3 Department of Population Health, QIMR Berghofer Medical Research Institute, Brisbane, Australia; 4 Dermatology Research Centre, The University of Queensland Diamantina Institute, The University of Queensland, Brisbane, Australia

**Keywords:** Bowen disease, dermatological surgery, surgical margins, nonmelanoma skin cancer

## Abstract

**Background:**

One common treatment for Bowen disease (BD) is surgical excision, but there is no international consensus on the appropriate surgical margins.

**Objectives:**

This study examined what factors affect the rate of incomplete excision of BD.

**Methods:**

Clinicopathological data potentially linked to surgical outcome (complete or incomplete excision) were retrospectively collected from medical and histopathological records on all surgically excised BD lesions diagnosed at Sahlgrenska University Hospital in Gothenburg, Sweden during 2014–2015. Data were analyzed with two definitions of incomplete excision: less strict (ie, BD present at the surgical margin) and strict (ie, dysplasia present at the surgical margin).

**Results:**

In total, 463 BD lesions among 408 patients were included. With the less strict definition, 3 factors were associated with significantly higher rates of incomplete excision: surgical margins <3 mm, a less experienced surgeon, and use of punch biopsy excision. The same factors plus a tumor location on the head and neck area or upper extremities were associated with significantly higher rates of incomplete excision using the strict definition. After adjustment for confounders, less experience was independently associated with incomplete excision using the less strict definition, whereas less experience and location on the head and neck area or upper extremities were independently associated with incomplete excision using the strict definition. Surgeon specialty was not associated with incomplete excision regardless of the definition.

**Conclusions:**

When removing BD surgically, an elliptical excision with surgical margins ≥3 mm carried out by an experienced surgeon should be recommended. Surgical margins may need to be adjusted depending on body site.

## Introduction

Bowen disease (BD), or squamous cell carcinoma (SCC) in situ, is a lesion with keratinocytic dysplasia restricted to the epidermis. The observed number of cases of BD has increased markedly in Sweden over the past few decades. In 2019, 13,782 cases of BD were reported to the Swedish National Board of Health and Welfare, as compared to 3,816 cases reported in 1999 [1]. The risk for BD to develop into invasive SCC has been estimated to be 3%–5% [2,3]. Treatment options for BD include photodynamic therapy, cryotherapy, curettage, 5% 5-fluorouracil cream, 5% imiquimod cream, ablative laser therapy, radiotherapy, and, of course, surgical excision [4,5]. No single treatment has been shown to be superior to any other and there is a lack of studies on treatment effects, especially regarding surgery [6,7].

Along with the rapid increase of skin cancer cases comes increased health costs and morbidity for the patients [8]. One way to decrease the rising costs is to reduce the number of incomplete excisions of keratinocyte cancer. Surgical excision of BD and skin cancers in general is carried out by surgeons of several specialties, including dermatologists, general practitioners (GPs), plastic surgeons, general surgeons, and otorhinolaryngologists. When surgery is considered to be the best treatment alternative, we must provide the surgeon with the right conditions, such as knowledge of optimal safety margins and information about potential risk factors for incomplete excision. Such factors could include lesion size, body site and excisional method [9–11].

There is no international consensus on the most appropriate surgical margins for BD. In fact, surgical margins are not mentioned in the European or Swedish clinical practice guidelines [12,13]. American guidelines advise using a 4–6 mm margin for low-risk SCC including BD [14], while French guidelines recommend surgical excision for BD with a “minimal margin”, but do not specify what this margin should be [5, 15].

Multiple studies compared incomplete excision rates of SCC in different settings, with rates ranging from 3.9% to 17.6% [15–18]. A study on wide local excisions of histopathologically proven SCCs included a subgroup of 514 BD lesions with an incomplete excision rate of 5.2% [19]. However, to our knowledge, only one previous study specifically analyzed risk factors for the incomplete excision of BD [10]. This Dutch study included 86 BD lesions excised with varying surgical margins, resulting in a 17.7% incomplete excision rate. The authors concluded that smaller excision margins seemed to be the most crucial risk factor. Furthermore, the study showed that incomplete excision rates were lower for dermatologists than for GPs and other specialists in secondary care.

The aim of this study was to further evaluate the clinicopathological risk factors for incomplete excision of BD in a larger series of patients.

## Materials and Methods

In this retrospective, observational study, we reviewed medical records and histopathological reports from all consecutive cases of excised and histopathologically confirmed BD lesions diagnosed at the Department of Pathology at Sahlgrenska University Hospital in Gothenburg, Sweden between January 1, 2014 and December 31, 2015. Data collection was approved by the regional ethical review board in Gothenburg.

The study included only histopathologically verified cases of BD managed by excisional biopsy. No clinical follow-up of the study participants was performed. The study participants were residents of the hospital catchment area in western Sweden, meaning that most would have been Caucasians with fair to medium skin types, although skin type was not consistently reported in the medical records [20]. The exclusion criteria were: BD treated with other methods, misclassified lesions (eg, BD in combination with microinvasive or invasive SCC, or BD found within or connected to other types of skin cancer), inadequate clinicopathological data, lack of data concerning clear margins, and BD located in the genital area or on mucous membranes. The latter type of BD is often HPV-induced and thus more difficult to treat [21].

A number of variables potentially linked to surgical outcome were obtained from medical records and histopathological reports. The clinicopathological parameters analyzed included patient age and sex, immunosuppression, tumor size and location, medical specialty and experience of the physician carrying out the surgery (specialist vs. resident), suspected diagnosis before excision, whether a preoperative biopsy was performed, the excisional biopsy method, the surgical margin (mm) as measured by the physician carrying out the excision, and whether clear margins were achieved. Data regarding complete removal were only collected from existing histopathology reports; the histopathology slides were not reviewed again.

When analyzing the data, we set up a number of criteria to ensure that the evaluations were made as objectively as possible. If re-excisions were carried out on the same lesion, only data from the first excision were included concerning the surgical margin and whether or not the margins were clear. If different tumor sizes were described in the medical records, the size of the tumor mentioned on the day of surgery was used. Excisions were interpreted as complete even if the distance to the resection margin was very limited. Since BD lesions can sometimes be isolated and are sometimes located within areas of chronically sun-damaged skin, two different definitions of an incomplete excision were applied in separate analyses. Incomplete excision according to the *less strict* definition implied the presence of BD or severe keratinocytic dysplasia at the border, while the *strict* definition of an incomplete excision was the presence of any degree of keratinocytic dysplasia at the specimen border.

### Statistical Analyses

Data were analyzed using version 3.0.3 of R (The R Foundation for Statistical Computing, Vienna, Austria). Fisher’s exact test was used to compare proportions, Wilcoxon’s rank sum test was used for two-sample tests, and the Kruskal-Wallis test was used to compare three or more groups. A multiple logistic regression analysis adjusted for possible confounders was performed to identify risk factors that were independently associated with incomplete excision. Outcome variables according to the strict and less strict definitions of incomplete excision were set separately, adjusting the logistic regression model for all risk factors significant on univariate analysis. P values of <0.05 were considered significant.

## Results

In total, 788 cases of surgically treated BD in 708 patients were found in the pathology register during the study period. After manual review of medical records and histopathological reports, 463 lesions in 408 patients matched the study inclusion criteria. Lesions were excluded due to treatment with topical drugs or other methods (n = 230), misclassification (n = 45), location on mucous membranes (n = 34), inadequate clinicopathological data (n = 10), and lack of data concerning clear margins (n = 6).

Slightly more than half of the patients were women (56.0%). The patients were generally elderly, with a median age of 77.6 years (range, 40–98 years). Among patients with known immune status (n = 270), 14.1% were immunosuppressed, and this group mainly consisted of organ transplant recipients. The median lesion diameter was 10.0 mm (range, 1–55 mm). A preoperative biopsy was taken in 191 cases (41.3%).

Clinicopathological features of excised lesions that may have influenced the incomplete excision rate are analyzed in Table 1. Lesions were most commonly excised by dermatologists (43.6%), followed by otorhinolaryngologists (16.8%), plastic surgeons (15.1%), general surgeons (13.0%), GPs (8.0%), and others (3.5%). The experience of the surgeon who performed the excision was unknown for 49 tumors, while for the remaining 414 tumors excision was done by a specialist in 87.0% or by a resident in 13.0% of the cases. The surgical margin was only reported in 215 cases (46.4%). The most common tumor location was the head and neck area (52.8%), followed by the trunk (21.6%). An elliptic excision was used to excise 417 (95.0%) of the 439 lesions with a known excisional biopsy technique.

There was a significant variation in the median diameter of excised tumors between the various medical specialties (P <0.001). GPs excised smaller lesions (median diameter, 6 mm), while plastic surgeons excised larger lesions (median diameter, 13 mm).

The suspected diagnosis was specified in the medical records and/or histopathological reports in 416 cases (89.8%). The diagnostic accuracy was low (21.6%) when only the main diagnosis was considered and increased slightly when differential diagnoses were also included (32.2%). After BD, the most commonly suspected main diagnosis was another keratinocytic tumor: basal cell carcinoma (BCC, 31.8%), followed by SCC (19.7%) and actinic keratosis (10.5%).

To understand which clinicopathological factors lead to incomplete excision, these variables were assessed using two definitions of incomplete excision, as discussed below and in Table 1.

### Less Strict Definition of Incomplete Excision

The less strict definition of incomplete excision produced a lower overall rate of incomplete excision (12.8%) than did the strict definition (see below). There were no significant differences in incomplete excision rates between surgeons with different specialties (P = .16), but specialists had a significantly lower incomplete excision rate than residents (10.8% vs. 25.9%, P = .004).

A significant difference in incomplete excision rates was seen regarding surgical margins, with a lower rate for a surgical margin of 3–5 mm than for <3 mm (8.7% vs. 31.6%, P = .009). No significant difference in incomplete excision rates was seen between cases with and without data on surgical margins (P = 0.26). Lesions on the trunk were incompletely excised in 7.0% of cases, while lesions in the head and neck area were most frequently incompletely excised (15.2%). Nevertheless, there were no significant differences in these rates.

The median tumor diameter was 10.0 mm for incompletely excised BD and 8.0 mm for completely excised lesions, but the difference was not significant (P = .86). There was also no significant difference in incomplete excision rates between tumors with diameter >10 mm and those with diameter ≤10 mm (P = .57).

A significant difference in incomplete excision rates was again seen when comparing excisional biopsy methods. Incomplete elliptic excisions were less common than incomplete punch biopsy excisions (10.0% vs. 36.0%, P = .002). A preoperative biopsy did not affect the incomplete excision rates. Incomplete excision occurred in 9.9% and 14.7% of the cases with and without a preoperative biopsy, respectively (P = 0.16).

According to an adjusted logistic regression analysis using the less strict definition, only a less experienced surgeon (resident vs. specialist) remained a risk factor for incomplete excision, with an odds ratio (OR) of 4.30 (95% CI, 1.54- 11.97; P = .005). In the adjusted analysis, we included the other significant variables from our univariate analysis (surgical margins and excisional biopsy method) as confounders.

### Strict Definition of Incomplete Excision

According to the strict definition of incomplete excision, 23.8% of all lesions were incompletely excised. There were no significant differences with regard to the surgeon’s specialty (P = .21) (Table 1). However, physician experience had a significant effect on the incomplete excision rate, with specialists outperforming residents (21.1% vs. 40.7%, P = .003).

Tumors excised with surgical margins <3 mm were incompletely removed in 47.4% of the cases, as compared to 20.4% when surgical margins of 3–5 mm were applied; this was a statistically significant difference (P = .018). There was no significant difference in incomplete excision rate between cases with and without data on surgical margins (P = .66).

Body site also significantly affected the surgical outcome (P < .001). In individual comparisons between body sites, excisions in the head and neck area were more frequently incomplete than excisions on the trunk (P < .001) and lower extremities (P = .046). Lesions on the upper extremities were also more frequently incompletely excised than lesions on the trunk (P < .001) and lower extremities (P = .032).

There was no significant difference between the median tumor diameter of incompletely and completely excised BD lesions, which was 10.0 mm in both groups (P = .46). There was also no significant difference in the incomplete excision rate between tumors >10 mm and those ≤10 mm in diameter (P = .24).

Although a punch biopsy excision was only used in 22 cases, this method had a significantly higher incomplete excision rate (45.0%) than did elliptic excision (21.0%, P = .016). As with the less strict definition, having undergone a preoperative biopsy did not affect the incomplete excision rate. Incomplete excisions were observed in 21.4% of cases with a preoperative biopsy and 26.1% of cases without one (P = .18).

According to the strict definition of incomplete excision, the adjusted logistic regression analysis yielded two independent risk factors for incomplete excision. One risk factor was a less experienced surgeon (resident vs. specialist), with an OR of 2.76 (95% CI, 1.25–6.10; P = .012). The other risk factor was a lesion location in the head and neck area or on the upper extremities (OR =3.15; 95% CI, 1.26–7.84; P = .014). In the adjusted analysis, all the significant variables from our univariate analysis were included, ie, surgical margins, excisional biopsy method, less experienced surgeon and body site.

## Discussion

We performed a large, retrospective study of surgery for BD. Using the less strict definition of incomplete excision, we found 3 parameters that were significantly associated with incomplete excision: surgical margins <3 mm, less experienced surgeon, and use of a punch biopsy excision. In addition to these parameters, a tumor location in the head and neck area or on the upper extremities was also a significant risk factor for incomplete excision according to the strict definition. For both definitions, a less experienced surgeon was independently associated with incomplete excision according to logistic regression, while a tumor location in the head and neck area or on the upper extremities was also independently associated with incomplete excision using the strict definition.

Although BD is often found on severely sun-damaged skin, it can also appear on otherwise healthy skin without sun damage. We therefore chose to analyze our data according to two separate definitions of incomplete excision. With the less strict definition, only 11.9% of the lesions were incompletely excised, which was slightly lower than the 17.7% rate reported by Westers-Attema et al (10). However, according to the strict definition, the overall incomplete excision rate was higher in our study (23.8%). Unfortunately, the article by Westers-Attema et al did not include a clear definition of what was considered an incomplete excision [10].

Our study did not show any significant difference in incomplete excision rates between surgeons from different medical specialties. This result differs from the study by Westers-Attema et al, in which dermatologists had an incomplete excision rate of only 8.8% while other specialties showed significantly higher rates (eg, 33.3% for GPs and 54.5% for plastic surgeons). However, this difference did not remain significant in the multivariate analysis [10]. Other studies on the successful management of keratinocyte cancers, especially those focusing on BCC, showed similar trends [9, 22–24]. In our study, the surgeons’ experience seemed to be of greater importance than their medical specialty. However, surgical experience was not measured in years or number of surgeries performed, but only if surgery was performed by residents or specialists. It is also worth noting that plastic surgeons performed surgery more often on lesions located in the head and neck area, ie, an area where surgery is considered more difficult to perform. Choosing adequate surgical margins for skin tumors is a balancing act. The surgeon needs to ensure clear margins in the excised tissue, but also wants to avoid the increased risk of hemorrhage and postoperative infection and the disfiguring scar of a larger excision. As BD is a noninvasive tumor with a relatively low risk of progressing into invasive SCC, it is desirable to minimize the surgical margin. Westers-Attema et al recommended a 5-mm margin to achieve complete excision and to avoid re-excision or additional noninvasive treatment [10]. According to our data, an elliptical excision with margins of at least 3 mm should be recommended. This could be adjusted depending on body site. Tumors located in the head and neck area or on the upper extremities were more often incompletely excised according to the strict definition. The fact that dysplasia was more often found at the specimen border in these cases may be explained by the fact that these areas, to a greater extent, contain chronically sun-damaged skin.

The present study is one of the largest on this topic to date, with 463 included cases. However, the retrospective design is a clear limitation, since not all data were available in the medical records and histopathology reports. For example, there was a great lack of data on immunosuppression. We were also not able to elucidate why surgery was chosen instead of treatment with other medical or destructive alternatives. However, the most common differential diagnoses were other keratinocyte cancers such as BCC and invasive SCC, which might explain why surgery was chosen. Furthermore, the diagnostic accuracy among excised BD lesions was low, with the correct diagnosis among the differential diagnoses only specified in less than a third of the cases. This could indicate that cases selected to be surgically excised differed from the most characteristic BD cases, where other non-surgical treatment is often chosen. Importantly, our strict definition of incomplete excision may not be relevant for body sites with extensive actinic damage since even subclinical dysplasia can be present in widespread areas. In such locations, dysplasia at the surgical border may either represent coincidental field cancerization or a true incomplete excision of the BD lesion. However, we had no means to differentiate between these two in our study.

European guidelines recommend performing surgical excision (or at least a preoperative biopsy followed by histopathology) when there is uncertainty about invasiveness, ie, to differ between an in situ tumor and early invasive SCC [12]. In our study, surgery was used in 41.3% of the cases in which a preoperative biopsy had already confirmed the diagnosis of BD. These findings may also indicate that the clinical and dermoscopic characteristics of BD treated by excision may not be representative of all BD lesions. Swedish guidelines state that excision of facial BD may be preferred to confirm complete removal [13]. In fact, more than half of the cases in this study were BD located in the head and neck area.

## Conclusions

When surgery was considered the best treatment method for BD, greater physician experience and the use of an elliptical excision with surgical margins of at least 3 mm were associated with lower incomplete excision rates. When a strict definition of incomplete excision was used, a tumor location on the trunk or lower extremities was also associated with lower incomplete excision rates. Based on these results, our recommendation is to use elliptic excision with at least a 3-mm excision margin when choosing surgery for treating BD. For lesions on chronically sun-damaged skin, the consequences of leaving certain degrees of dysplasia at the specimen border should be weighed against the consequences of re-excision.

## Figures and Tables

**Table 1:** Clinicopathological Factors Associated with Incomplete Excision for 463 Bowen Disease Lesions

Clinicopathological Factor	Tumors, n (%)	Incomplete Excision			
		Less Strict		Strict	
		*n* (%)	*P*	n (%)	*P*
Specialty			.16		.21
Dermatologist	202 (43.6)	24 (11.9)		44 (21.8)	
Otorhinolaryngologist	78 (16.8)	8 (10.3)		19 (24.4)	
Plastic surgeon	70 (15.1)	6 (8.6)		12 (17.1)	
General surgeon	60 (13.0)	9 (15.0)		17(28.3)	
General practitioner	37 (8.0)	10 (27.0)		11 (29.7)	
Others	16 (3.5)	2 (12.5)		7 (43.7)	
Missing	0				
Experience			.004		.003
Specialist	360 (87.0)	39 (10.8)		76 (21.1)	
Resident	54 (13.0)	14 (25.9)		22 (40.7)	
Missing	49				
Surgical margins			.009		.018
<3 mm	19 (8.8)	6 (31.6)		9 (47.4)	
3–5 mm	196 (91.2)	17 (8.7)		40 (20.4)	
Missing	248				
Body site			.22		< .001
Head & neck	244 (52.8)	37 (15.2)		71 (29.1)	
Trunk	100 (21.6)	7 (7.0)		10 (10.0)	
Upper extremities	60 (13.0)	8 (13.3)		20 (33.3)	
Lower extremities	58 (12.6)	7 (12.1)		9 (15.6)	
Missing	1				
Tumor diameter			.57		.24
≤10 mm	229 (71.3)	26 (11.4)		47 (20.5)	
>10 mm	92 (28.7)	13 (14.1)		25 (27.2)	
Missing	142				
Excisional biopsy method			.002		.016
Elliptic	417 (95.0)	43 (10.0)		89 (21.0)	
Punch	22 (5.0)	8 (36.0)		10 (45.0)	
Missing	24				
